# The Effects of Digital-Based Cognitive Behavioral Therapy for Insomnia (CBT-I) on Sleep Quality in Shift Workers: A Scoping Review

**DOI:** 10.7759/cureus.85086

**Published:** 2025-05-30

**Authors:** Taylor Yuska, Aleya DeVries, Melina Kanelos, Daniela Delphus, Katie Toperzer, Kaitlyn O'Malley, Sammy Katerji, Priyal Desai, Kim Pedrigal, Romon Thach, Pei-Fen Li

**Affiliations:** 1 College of Osteopathic Medicine, Nova Southeastern University Dr. Kiran C. Patel College of Osteopathic Medicine, Clearwater, USA; 2 Department of Couple and Family Therapy, Nova Southeastern University Dr. Kiran C. Patel College of Osteopathic Medicine, Fort Lauderdale, USA

**Keywords:** cognitive behavioral therapy for insomnia (cbt-i), digital health technology, insomnia, insomnia severity index, mobile health app, pittsburgh sleep quality index (psqi), shift work, shift work sleep disorders (swsd), sleep quality

## Abstract

Insomnia, characterized by difficulty falling asleep or staying asleep, is prevalent among shift workers due to irregular work hours. Cognitive behavioral therapy for insomnia (CBT-I) is the first-line treatment for alleviating insomnia symptoms; however, accessibility of CBT-I for shift workers is limited. To increase the user access and usefulness of CBT-I, digital CBT-I (dCBT-I) has attracted growing clinical and research interest in the field. This scoping review examines the effects of dCBT-I on improving sleep quality among shift workers. A comprehensive literature search across multiple databases identified 11 primary studies from 2014 to 2024, encompassing various shift-working populations. The findings were synthesized into themes, including the impacts of dCBT-I on sleep quality, sleep hygiene, objective sleep data, anxiety, depression, quality of life, and user feedback. All studies showed improved sleep quality after the implementation of dCBT-I, except for one. Two studies suggest that dCBT-I is equally effective as in-person delivery of CBT-I in improving sleep quality among shift workers. Per user feedback, incorporating individualized feedback and more customization options in dCBT-I would improve engagement and adherence to the intervention. Collectively, the findings in this review suggest that dCBT-I is an accessible and effective alternative to traditionally delivered CBT-I for improving sleep quality in shift workers.

## Introduction and background

According to the International Classification of Sleep Disorders, insomnia is defined as a persistent difficulty with sleep initiation or maintenance, despite adequate opportunity for sleep, that leads to daytime impairment [1,2]. Insomnia affects about 10%-20% of the general adult population, with around 50% of those cases being chronic [3]. It is characterized by fatigue, irritability, malaise, impaired function, attention, and memory [4]. Research shows a higher pervasiveness of sleep disorders in shift workers in contrast to regular daytime workers [5]. Approximately 32% of night workers, 10% of daytime workers, and 8%-26% of shift workers with rotating schedules experience shift work disorder [6].

Shift work disorder is described as complaints of insomnia and excessive daytime sleepiness, often due to scheduled work hours coinciding with typical sleep times [7]. Shift work disorder can incur an unfavorable impact on sleep quality and quality of life (QoL), leading to diminished health [8]. Globally, nearly one in five employees work non-traditional hours outside the typical 9 AM-5 PM schedule [9]. Shift workers encompass a wide range of professions, such as healthcare workers, air traffic controllers, first responders, security services, energy sector employees, and public transit workers, to name a few, all of whom must remain alert during their shifts. In addition to a decrease in QoL for the individual, shift work disorder leads to impaired work performance, increased mistakes, and a higher occurrence of accidents [10]. The consequences of shift work disorder are costly, both monetarily and in terms of public safety. For instance, night shift work increases driver sleepiness, which is associated with a four- to sixfold increase in motor vehicle accidents or near-accidents [11]. Additionally, inadequate sleep among shift workers in the United States costs approximately $30-$40 billion each year in healthcare costs [12].

Shift work disorder has been treated with modafinil and blue light therapy in the past. Despite some modest improvements using modafinil to modulate neurotransmitter levels, many patients continued to have excessive sleepiness and decreased vigilance while working at night [13]. Among non-pharmacological treatments for shift work disorder, blue light therapy has been used to regulate circadian rhythm and has shown positive effects on sleepiness while driving [14]. However, a recent study suggests blue light therapy did not significantly reduce sleepiness or improve alertness during shifts [15]. In contrast, behavioral therapies have shown greater promise. Insomnia experienced by shift workers has been effectively treated using face-to-face behavioral therapy that restricted the sleep window of participants [16]. There is evidence that cognitive behavioral therapy for insomnia (CBT-I) has superior results in treating insomnia in people with major depressive disorder than using antidepressant therapy alone [17]. Among psychotherapies for insomnia, CBT-I has the strongest evidence base and is the preferred intervention for chronic insomnia according to the American College of Physicians [18].

CBT-I is a therapy that addresses and mitigates difficulty with initiating and maintaining sleep. An established theory of insomnia development is the "3P" model, which sets forth that chronic insomnia could be the result of an aggregate of predisposing factors, a precipitating event, and perpetuating factors [19]. CBT-I primarily focuses on attenuating the perpetuating factors, such as learned negative associations with sleep that advance the development of insomnia [20]. This is accomplished using the following four pillars: sleep restriction therapy, stimulus control therapy, sleep hygiene, and cognitive therapy [20]. In a study by Takano et al. [21], a single 90-minute CBT-I session significantly improved sleep quality and psychological distress in shift workers at a two-month follow-up compared to a control group. Additionally, the benefits of CBT-I have been found to last for at least six months following treatment for shift workers with chronic insomnia [22].

CBT-I is an effective treatment option. However, it requires the patient to take part in face-to-face, in-person sessions for multiple visits [23]. Due to shift workers’ irregular schedules, the normal operating hours of standard clinics may limit access to in-person care for shift workers. Telemedicine-based CBT-I, involving real-time sessions with a clinician via video or phone, has been shown to be equally effective in improving sleep quality when compared to face-to-face CBT-I [24]. Building on the success of telemedicine-based CBT-I, there has been increased interest in digital CBT-I (dCBT-I), which involves self-guided web- or app-based programs that deliver the core components of CBT-I without live clinician sessions. For those who suffer from insomnia, dCBT-I has been shown to have promising results when used across multiple populations of diverse socioeconomic status, race, and gender [25]. Given its effectiveness, dCBT-I may offer a more accessible and cost-effective solution for shift workers compared to traditional in-person therapy or pharmacological treatment [26].

Traditional CBT-I has been shown to be successful in treating insomnia among shift workers. With growing evidence supporting the usefulness of dCBT-I for insomnia, this presents the question: What are the effects of dCBT-I in shift workers to improve their sleep quality? This current study’s objective is to provide a scoping review of the current findings on the effectiveness of dCBT-I as a method to improve sleep quality in shift workers and to recognize prospective challenges associated with this newer modality of delivering CBT-I.

## Review

Methods

The methods for this scoping review were based on the Arksey and O’Malley framework: identifying the research question, searching for relevant studies, study selection, data charting, and finally, summarizing, synthesizing, and reporting the results [27].

Eligibility Criteria

This scoping review included primary experimental studies (including randomized controlled trials (RCTs) and non-RCTs) and observational studies (including analytical observational studies, case-control studies, and analytical cross-sectional studies) published in English between 2014 and 2024. Systematic and scoping reviews were excluded. Eligible participants were adults (18 years and older) engaged in shift work who had utilized dCBT-I. The review excluded participants under 18 years old, in-person delivered CBT-I, pharmacological approaches, and other non-digital psychotherapeutic approaches. The review question “What are the effects of digital CBT-I on shift workers’ sleep quality” guided the search.

Search Strategy

An initial search using the databases Embase, Ovid MEDLINE, and Web of Science identified articles by a preliminary search of titles and abstracts. Table 1 shows the search terms used for Embase. The same search terms were translated for use in Ovid MEDLINE and Web of Science.

**Table 1:** Iterative development of Boolean search terms used in Embase. Illustration of the iterative refinement process that led to the final Boolean search strategy presented in query #7. Each search was performed individually, and the number of results reflects the output of each distinct query; the outputs are not intended to be summed.

Search number	Search query	Number of results
#1	'shift work sleep disorder'/exp OR 'shift work'/exp	7,793
#2	'shift work*':ab,ti,kw OR 'shiftwork*':ab,ti,kw OR 'sleep quality':ab,ti,kw OR 'sleep improve*':ab,ti,kw OR 'shift*':ab,ti,kw	635,633
#3	'cognitive behavioral therapy'/exp	33,064
#4	'cbt*':ab,ti,kw OR 'digital*':ab,ti,kw OR 'online':ab,ti,kw OR 'telehealth':ab,ti,kw OR 'internet*':ab,ti,kw OR 'smartphone*':ab,ti,kw OR 'cognitive behavior* therap*':ab,ti,kw OR 'cognitive behavior* therap* for insomnia*':ab,ti,kw OR 'cbt-i*':ab,ti,kw OR 'digital* cognitive behavior* therap*':ab,ti,kw	792,204
#5	#3 OR #4	808,353
#6	#1 AND #2	6,053
#7	#5 AND #6	387

Study Selection

**Figure 1:**
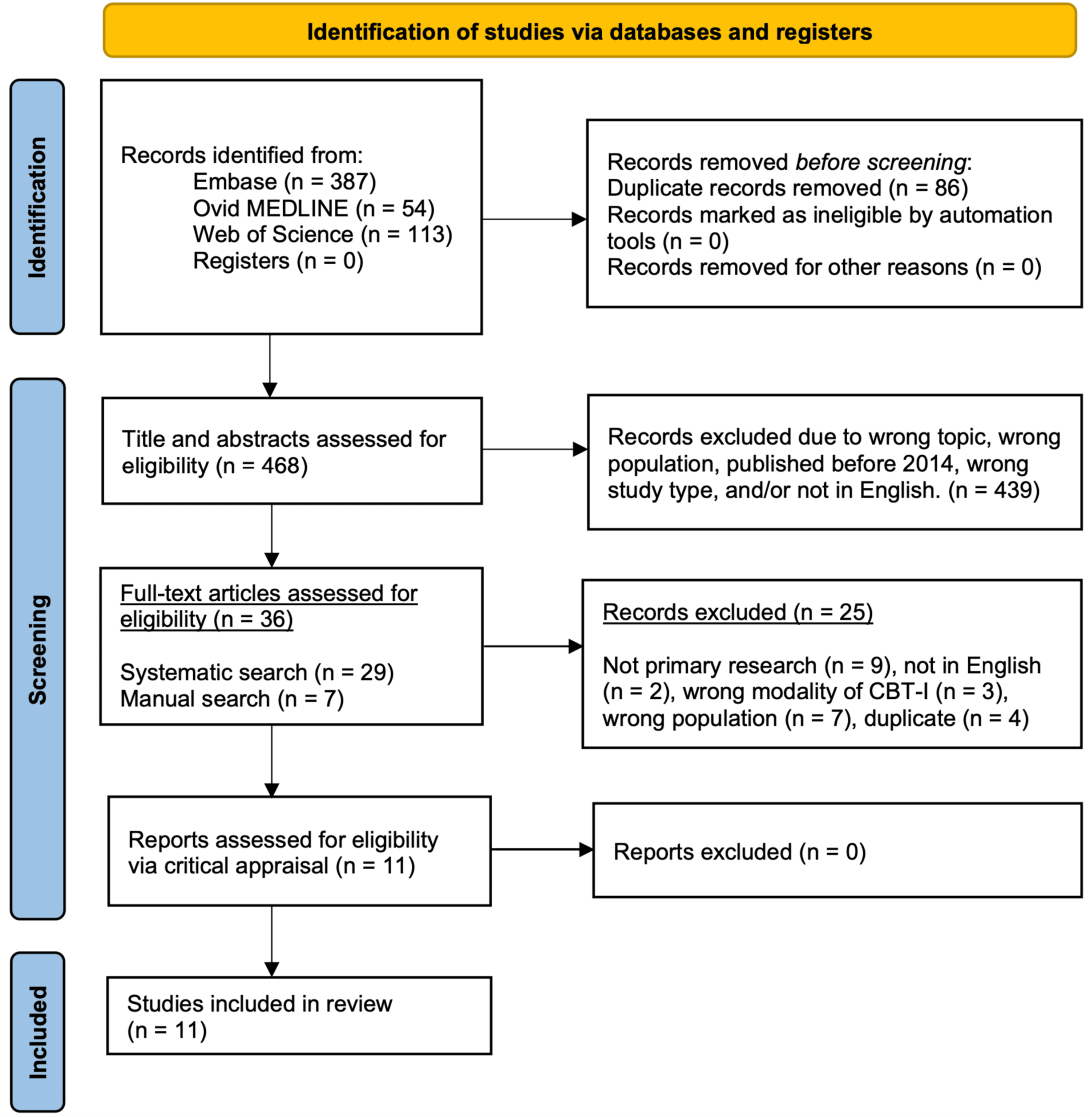
Preferred Reporting Items for Systematic Reviews and Meta-Analyses (PRISMA) diagram of the search strategy. CBT-I: cognitive behavioral therapy for insomnia

The study selection was completed by screening abstracts and full-text articles against the inclusion criteria. An additional seven relevant articles were identified through a manual search of Google Scholar and PubMed. Finally, a critical appraisal of the articles was conducted using the Joanna Briggs Institute (JBI) Critical Appraisal Tool [28,29]. The study selection process is detailed in a Preferred Reporting Items for Systematic Reviews and Meta-Analyses (PRISMA) diagram (Figure 1).

Data Charting and Extraction Process

Data from the final 11 studies were extracted and charted in Excel (Microsoft Corp., Redmond, WA, US). The extracted data included the study aim, populations studied, study design, methods, sample size, study limitations, and key findings.

Results

**Figure 2:**
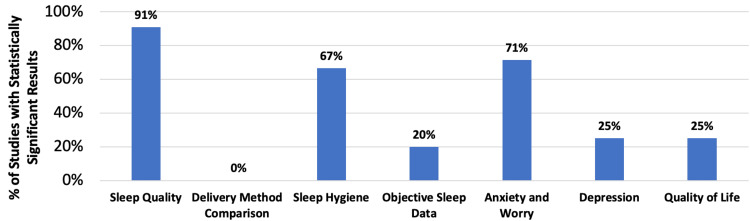
The percentage of selected studies that reported statistically significant findings for key themes. Sleep quality: 91% of studies that measured subjective sleep quality and insomnia severity (N = 11) found significant improvement [30-40]. Delivery method comparison: 0% of studies comparing dCBT-I with in-person CBT-I (N = 2) demonstrated significant differences in sleep quality outcomes between delivery methods [33,34]. Sleep hygiene: 67% of studies that measured sleep hygiene (N = 3) found significant improvements in sleep hygiene habits [30,37,40]. Objective sleep data: 20% of studies that collected objective sleep data (N = 5) found significant improvement in actinography-based metrics, such as total sleep time [31,34,36,38,39]. Anxiety and worry: 71% of studies that measured anxiety and worry symptoms (N = 7) found significant improvement [31,34,36-40]. Depression: 60% of studies that measured depression symptoms (N = 5) found significant improvement [31,34,37,38,40]. Quality of life: 25% of studies that measured quality of life (N = 4) found significant improvement [33,34,36,39]. CBT-I: cognitive behavioral therapy for insomnia; dCBT-I: digital cognitive behavioral therapy for insomnia

All studies in this scoping review, except Oftedal et al. [30], showed an improvement in sleep quality by using dCBT-I interventions in shift workers. All studies focused on shift workers. However, their study populations differed slightly. Of the 11 studies, four focused on healthcare workers (primarily nurses), four on shift workers generally, one on pilots, and one on bus drivers, and one included healthcare workers and other industries. The majority of studies were pilot studies or early efficacy trials and had relatively small sample sizes with substantial attrition. Participant numbers ranged from 13 to 83, with one outlier having 502. All studies were published between the years 2014 and 2024. Of the data collected, the evidence was categorized into impacts on sleep quality, sleep hygiene, objective sleep measures, anxiety, depression, QoL, and user feedback. The 11 articles included in this scoping review are summarized in table format (Table 2). Additionally, a bar chart summarizes the percentage of selected studies that reported statistically significant findings for key themes (Figure 2).

**Table 2:** Summary of the final 11 articles. CBT-I: cognitive behavioral therapy for insomnia; dCBT-I: digital cognitive behavioral therapy for insomnia; ISI: insomnia severity index; SWD: shift work disorder

Authors	Purpose	Study design	Population and sample size	Methods	Limitations	Key findings
Lee et al. (2014) [31]	Evaluate the efficacy of home-based dCBT-I sleep training	Longitudinal, pre-/post-intervention study	Night shift nurses (N = 21)	Active control phase followed by home-based dCBT-I intervention, both four weeks. Wrist actigraphy and questionnaires were completed at baseline and post-interventions	Small sample size with no control group. Pilot study design	Significant improvement in subjective sleep quality, anxiety, and depression symptoms reported. No significant improvements in objective sleep quality measures reported
van Drongelen et al. (2014) [32]	Explore dCBT-I mobile app (mHealth) designed to improve sleep in airline pilots	Randomized controlled trial	Airline pilots (N = 502)	Randomized trial split into two groups: (1) sleep hygiene control and (2) dCBT-I sleep app, mHealth (given specific sleep and health advice). Variables were assessed at baseline, then at three- and six-month post-intervention	Data were self-reported. Participation was voluntary, so potential for selection bias. Adherence to app usage was limited	Sleep quality significantly improved in the dCBT-I group
Oftedal et al. (2019) [30]	Evaluate the effectiveness of an app-based dCBT-I aimed at the sleep and health of shift workers	Pilot randomized controlled trial	Shift workers (N = 40)	Randomized trial split into two groups: (1) waitlist control and (2) app-based dCBT-I intervention (targeting sleep, physical activity, and diet). Measurements were taken over four weeks	Pilot study, small sample size, short intervention time. Issues with user engagement. Only self-reported measures	No significant improvement in sleep quality or sleep hygiene between groups. Users expressed dissatisfaction with the lack of customization of dCBT-I
Peter et al. (2019) [33]	Examine the efficacy of dCBT-I in comparison to face-to-face CBT-I among shift workers with SWD	Non-randomized comparative trial	Shift workers (N = 33)	Non-randomized trial compared four weeks of at-home dCBT-I delivered by email to six sessions of face-to-face CBT-I led by a therapist. Both groups completed sleep diaries. Measurements were taken at baseline and after treatment	Small sample size, no control group, no randomization. No long-term follow-up	Both dCBT-I and in-person CBT-I groups had significant improvements in sleep quality. No significant difference between groups
Järnefelt et al. (2020) [34]	Examine the efficacy of CBT-I and dCBT-I compared to a sleep hygiene intervention in shift workers	Randomized controlled trial	Shift workers with insomnia disorder (N = 83)	Randomized trial split into three groups: (1) group-based CBT-I, (2) dCBT-I, and (3) sleep hygiene control. Measurements were taken at baseline, after completion, and six months post-treatment	Small sample size. Few small differences between group-based CBT-I and sleep hygiene intervention groups. Inconsistent compliance	Sleep quality significantly improved in all groups with no significant difference between groups
Omeogu et al. (2020) [35]	Evaluate the effectiveness of app-based dCBT-I in decreasing insomnia among hospital nurses	Single-arm, non-randomized trial	Hospital nurses with moderate/severe insomnia (N = 17)	Participants used the dCBT-I app for six weeks with no external guidance. Sleep quality was assessed three times: (1) baseline, (2) three weeks, and (3) six weeks	Small sample size, no control group. Participants self-reported outcomes. There was a small amount of attrition	Significant decrease in insomnia severity at three and six weeks
Ito-Masui et al. (2023) [36]	Investigate the effects of physician-assisted, internet-based dCBT-I for healthcare shift workers	Single-arm, non-randomized trial	Shift workers in ICU/emergency department (N = 61)	Participants used physician-guided, internet-based dCBT-I for four weeks. This included personalized sleep advice. Fitness trackers provided monitoring along with self-reports	No control group, short duration of treatment, no long-term measurements. Non-randomized. Convenience sampling with financial incentives	Significant improvement in sleep duration, per wearable sensors. Significant improvement in sleep quality. Improved user retention rates with physician-led feedback
Murray et al. (2023) [37]	Evaluate the efficacy of mobile app dCBT-I (SleepSync), designed to improve sleep in shift workers	Pilot, single-arm, non-randomized trial	Shift workers (N = 27)	Participants used the SleepSync app to receive dCBT-I. The app provided personalized sleep guidance and tracking for two weeks. Self-reports were completed at baseline and post-trial	Small sample size, no control group, short duration of treatment. Gender and profession biases	Significant improvements in sleep quality and length, mood, and insomnia severity. Positive user feedback on usability and integration. No significant change in depression symptoms
Ell et al. (2024) [38]	Examine the effectiveness of the dCBT-I program (SleepCare) targeted toward nurses with SWD	Randomized controlled trial	Nurses diagnosed with SWD with insomnia (N = 46)	Randomized trial split into two groups: (1) waitlist control and (2) SleepCare dCBT-I sessions. Three assessments conducted: (1) baseline, (2) post-treatment, and (3) six months post-trial (dCBT-I group only)	Small sample size, use of waitlist group, variable shift schedules, not allowed to wear actigraphy devices at work	dCBT-I group had significantly decreased insomnia severity and depression. Stability at the six-month follow-up. Positive user feedback given on usability and individualized feedback
Järnefelt et al. (2024) [39]	Evaluate the implementation of digital sleep coaching using dCBT-I	Mixed-methods implementation study	Professional bus drivers from two Finnish companies (N = 30)	Participants separated into two dCBT-I groups based on ISI scores: (1) brief (one session) or (2) extended (four sessions). Workshops and surveys were conducted over the nine-month period	Small sample size, with most of the sample being men, challenges in communication, and conducted during the COVID-19 pandemic	Insomnia severity was significantly reduced. Worry about driving while sleepy was reduced. Flexibility of dCBT-I increased participation
Varma et al. (2024) [40]	Evaluate the efficacy of dCBT-I app (SleepSync) in managing sleep and cognitive fitness in defense workers	Single-arm, non-randomized trial	Shift workers from a Royal Australian Defense Force base (N = 13)	Participants used the dCBT-I app (SleepSync), which provided personalized sleep recommendations and informational resources (caffeine use, light exposure) for four weeks. Measurements were taken at baseline and post-treatment	Non-randomized trial, small sample size, lacked long-term follow-up, self-reported data	Statistically significant reduction in insomnia severity and improved sleep hygiene. No significant change in depression symptoms

Impact on Sleep Quality

All studies in this scoping review utilized validated measures of sleep quality, such as the insomnia severity index (ISI) and the Pittsburgh Sleep Quality Index (PSQI). Of the 11 studies, 10 studies reported an improvement in sleep quality, as evidenced by an improvement in the ISI or PQSI following dCBT-I intervention [30-40].

Seven studies utilizing the ISI found statistically significant decreases in ISI scores, indicating decreased insomnia severity [33-35,37-40]. Two of these studies used a combination of ISI, PQSI, and sleep efficiency to compare the outcomes of dCBT-I with in-person CBT-I [33,34]. While all treatment groups demonstrated significant improvement in sleep quality, no statistically significant differences were found between delivery methods. The lack of a significant difference between in-person CBT-I and dCBT-I suggests a similar, non-inferior therapeutic benefit in reducing insomnia severity.

Four studies used the PSQI to assess sleep quality [30-32,36]. Three of the four studies showed significant improvements in the global PSQI score, or components of the PSQI, following the implementation of dCBT-I [31,32,36]. However, one study found no significant difference, which reported an average PSQI score of 7.5 in the intervention group and 7.9 in the waitlist group [30]. Overall, the findings suggest that dCBT-I improves insomnia symptoms and sleep quality.

Impact on Sleep Hygiene

Ideal sleep hygiene involves behavioral and environmental strategies, such as keeping a regular sleep and wake schedule, engaging in regular physical activity, and limiting daytime naps [41]. Three studies collected data on sleep hygiene [30,37,40]. Murray et al. [37] and Varma et al. [40] showed significant decreases in the Sleep Hygiene Index (SHI), indicating better sleep hygiene habits following dCBT-I. However, Oftedal et al. [30] showed a non-significant difference when comparing the average SHI scores of the intervention group (M = 39.5) and the waitlist group (M = 36.4) [30]. Overall, the findings suggest that dCBT-I may contribute to the development of improved sleep hygiene habits.

Impact on Objective Sleep Data

Five studies used wrist actigraphy to obtain objective sleep data, such as sleep onset latency, wake after sleep onset, sleep efficiency, and total sleep duration [31,34,36,38,39]. Participants placed tracking devices on their non-dominant hands and wore them throughout the night to obtain data during their sleep. Four studies did not find a significant change in sleep onset latency, wake after sleep onset, and sleep efficiency with the implementation of dCBT-I [34,36,38,39]. However, the remaining study found a significant increase in the total sleep duration, with an average increase of 0.52 hours of additional sleep compared to baseline after dCBT-I [36]. Together, the studies suggest that dCBT-I does not have a significant impact on objective sleep measures.

Impact on Anxiety and Worry

Seven studies evaluated the impacts of dCBT-I on worry or anxiety at baseline and after the intervention [31,34,36-40]. Of these studies, five reported significant alleviation of worry or anxiety following dCBT-I intervention [31,34,37-39]. For example, Lee et al. [31], using the Standard Shiftwork Index (SSI), found a significant decrease in cognitive and somatic anxiety symptoms. Similarly, Järnefelt et al. [34], using the Penn State Worry Questionnaire (PSWQ), found that worry significantly decreased. Furthermore, Järnefelt et al. [39], found a significant decrease in worry about having an accident among bus drivers while sleepy.

Two studies used the State-Trait Anxiety Inventory (STAI) and produced mixed results. Ell et al.’s [38] study found a significant decrease in state anxiety but not trait anxiety, whereas Ito-Masui et al. [36] found no significant reduction in state or trait anxiety. Two studies using the Depression, Anxiety, Stress Scale-21 (DASS-21) also produced mixed results. Murray et al. [37] found a significant lessening of anxiety, while Varma et al. [40] failed to find a significant decline in anxiety. Overall, despite some variation across studies, the majority suggested that dCBT-I may effectively reduce anxiety and worry among shift workers.

Impact on Depression

To determine the impacts of the dCBT-I on depression, five studies used a variety of measurement tools to assess depression symptom levels at both pre- and post-intervention [31,34,37,38,40]. Ell et al. [38], using the Beck Depression Inventory (BDI-II), found a significant decline in depression symptoms following dCBT-I intervention. Järnefelt et al. [34], also using BDI-II, reported a significant reduction in depression; however, the interaction between treatment condition and time was not statistically significant, suggesting that the reduction in depression symptoms cannot be conclusively attributed to the intervention.

Lee et al. [31] used the Center for Epidemiologic Studies-Depression (CES-D) scale and found a significant reduction in depression symptoms following dCBT-I intervention. In contrast, Varma et al. [40] and Murray et al. [37], using DASS-21, reported trends toward lessening depression symptoms, although this reduction did not reach statistical significance. Overall, the collective evidence suggests that dCBT-I may relieve depression symptoms in shift workers, and variations in statistical significance were potentially caused by different study methods and measurement tools.

Impact on QoL

Four studies evaluated measures of wellness or QoL [33,34,36,39]. Peter et al. [33], using the World Health Organization Wellbeing Questionnaire, found that well-being significantly improved following dCBT-I intervention. Järnefelt et al. [34] found a trend toward improvement in mental health-related QoL using the RAND-36 Health Survey. Ito-Masui et al. [36] found no significant improvement in well-being scores except for morning energy, and Järnefelt et al. [39] observed no significant change in QoL as measured by the Euro-HIS-8 scale. Overall, the findings suggest that dCBT-I may support mild improvements in QoL and well-being. However, the evidence is inconclusive.

Feedback From Shift Workers Regarding dCBT-I

Several studies have reported on user satisfaction with dCBT-I, collecting feedback through various methods, including interviews, surveys, and app usage metrics. The primary strengths identified were the flexibility, usability, and accessibility of dCBT-I. For example, Ell et al. [38] found that 94.7% of respondents were satisfied or highly satisfied with the intervention and rated the technical feasibility as high or very high. Additionally, 89.5% of participants valued the flexibility of accessing the intervention regardless of location or time, and 73.7% found the intervention easy to integrate into their daily lives [38]. Similarly, Murray et al. [37] found that 82% of users found that it was easy to integrate the app into their daily routines. Järnefelt et al. [39] found that allowing users to choose the time and pace of participation facilitated the implementation of dCBT-I. Together, the findings suggest that flexibility and usability were key strengths of dCBT-I for shift workers.

Individualized feedback and tailored content were also identified as important facilitators for engagement. Ell et al. [38] highlighted that individualized guidance was among the most helpful aspects of the intervention. Three studies included some form of personalized feedback through the app, delivered by either a clinician or automated means [36-38]. The only study that used weekly individualized feedback from a sleep physician retained almost all participating members, losing only one participant due to insufficient data [36]. The remaining two studies utilized tailored guidance through automated algorithms [37,38]. In general, all three studies that used personalized feedback appeared to have higher retention rates overall. Together, these findings suggest that a more personalized experience, whether clinician-driven or AI-driven, may enhance engagement and compliance with dCBT-I.

A weakness of dCBT-I expressed by shift workers was the limited ability to customize the intervention to fit their individual needs. For example, the participants in Oftedal et al.’s study [30] reported that the intervention lacked customization in logging details and expressed a desire for a more tailored intervention with interactive features. Additionally, participants in Oftedal et al.’s study [30] noted that the handbook was too lengthy and difficult to engage with. Similarly, Järnefelt et al. [39] reported that overly abstract and generalized content hindered its practical application to the participants. In summary, these findings suggest that the lack of customization and interactivity of content is a weakness in several dCBT-I interventions.

Discussion

The retrieved studies in this scoping review were analyzed to determine the effects of dCBT-I on sleep quality in shift workers. Additionally, the analysis examined the impact of dCBT-I on sleep hygiene, objective sleep data, anxiety, depression, and QoL.

Previous research in general insomnia populations has demonstrated that dCBT-I is comparable in efficacy to traditional face-to-face CBT-I in decreasing insomnia severity [24]. This scoping review supports this conclusion in the shift work population. Two reviewed studies, Peter et al. [33] and Järnefelt et al. [34], directly compared a dCBT-I intervention with an in-person CBT-I intervention in shift workers. Both studies found that insomnia symptoms were reduced in both groups, with no statistically significant difference between the digital and face-to-face interventions. This finding suggests that dCBT-I is comparable in efficacy to traditional CBT-I for shift workers in decreasing insomnia severity. For shift workers who may have difficulty in scheduling traditional CBT-I, the findings in Järnefelt et al. [34] and Peter et al. [33] demonstrate that dCBT-I is an effective and practical alternative. dCBT-I is flexible and scalable, offering a promising and similarly effective treatment for shift workers who may have barriers to accessing in-person treatment.

Ten out of the 11 studies in this scoping review showed that dCBT-I improves sleep quality and insomnia symptoms in shift workers. Notably, Lee et al. [31] proposed that an increase in sleep duration does not lead to improved sleep quality. This finding suggests that dCBT-I could significantly improve subjective sleep quality symptoms without the need to increase the overall sleep duration [31]. This finding is especially valuable in the shift-working population due to their time constraints for sleep, long working hours, and subsequent sleep deprivation.

Some studies showed inconsistencies between self-reported and objective sleep data. While objective data of sleep latency and quality through wrist actigraphy showed no significant improvements, subjective insomnia symptoms showed improvement. Wrist actigraphy is an indirect measure of sleep, as it measures body movements to determine wake and sleep states [42]. Although cost-effective, it is not the most precise measure of sleep [36]. Future studies should explore using different objective techniques with greater precision, such as polysomnography, which can be used to directly measure brain electrical activity [42]. Using a more precise objective measure in combination with subjective measures may give less conflicting data. However, while polysomnography provides more accurate measurements of sleep, the lab environment may reduce sleep efficiency, potentially skewing results.

Beyond dCBT-I's efficacy in treating insomnia symptoms, dCBT-I may also secondarily improve emotional and physical well-being in shift workers. Overall, the findings from this scoping review suggest that dCBT-I can alleviate worry or anxiety symptoms [31,34,37-39]. However, the results were not uniform across studies. The inconsistency could be attributed to the nature and underlying focus of dCBT-I itself. As dCBT-I is targeted at changing individuals’ negative cognitive thoughts and beliefs about sleep, it is well suited to address cognitive symptoms of anxiety. In contrast, it may be less effective in addressing the physiological components of anxiety. The physiological components may require more physically oriented interventions, such as relaxation techniques. The mixed results identify the need for future research to examine how the nature of dCBT-I influences the different aspects of anxiety, such as cognitive and physical symptoms. Clarifying dCBT-I's effect on anxiety relief is particularly important in the shift work population due to many shift workers working in high-stress occupations.

Traditional CBT-I has been proven to be effective in treating insomnia and depression in people with major depressive disorder [17]. This scoping review aligns with the previous research and highlights that dCBT-I can also improve depressive symptoms in addition to improving insomnia symptoms in shift workers. This may reflect the bidirectional relationship between sleep and mood, as poor sleep can exacerbate depression and vice versa. Therefore, improving sleep may have a corollary benefit of reducing depression symptoms [43]. The data from several studies indicate that dCBT-I can help reduce the severity of depression by alleviating symptoms across domains such as mood, behavior, physical well-being, and cognition [31,34,38]. Given that depression is a leading cause of absenteeism among workers, dCBT-I’s ability to address depression may lead to increased productivity and overall well-being for shift workers [44].

Effective therapy for shift work sleep disorder should also improve the QoL of the shift worker, as a decrease in QoL in those with shift work disorder leads to impaired work performance, increased mistakes, and a higher occurrence of accidents [10]. This scoping review, in general, has highlighted the secondary physical and psychological benefits of dCBT-I intervention. The secondary reduction in depression and anxiety may improve the QoL in shift workers, enhance their work performance, and decrease work-related accidents [31,34,38].

Several studies in this review suggested that shift workers who receive personalized feedback, either clinician-driven or AI-driven, had better compliance and adherence to dCBT-I and, thus, are more likely to continue engaging in the therapy over time [30,36-39]. To enhance motivation and continued engagement, shift workers would benefit from individualized progress reports, interactive goal setting, and real-time feedback. These feedback mechanisms can reinforce positive behavior changes, similar to the real-time feedback shift workers would receive from traditional CBT-I. Without personalized feedback, adherence to dCBT-I decreases over time, which may reduce the effectiveness of the therapy. To maintain the accessibility and effectiveness of dCBT-I, future research should investigate which feedback models, such as clinician-driven or AI-driven, yield the most statistically significant compliance rates.

A number of studies analyzed within this scoping review present several limitations, including small sample sizes, the absence of participant blinding, non-randomization, and a lack of control variables. Additionally, inconsistencies in using the same measurement tools make it challenging to compare outcomes across studies. Potential limitations of this scoping review may be attributed to the search strategy. The inclusion criteria for the systematic search process did not specify the type of digital modalities of dCBT-I, resulting in various formats of digital delivery methods across studies. However, to address this limitation and increase the robustness of the scoping review process, the authors implemented two tiers of peer review and a final critical appraisal of all selected articles using the JBI quasi-experimental or RCT checklist.

Future studies should include larger sample sizes with consistent, long-term follow-up to assess the sustainability of dCBT-I’s effects. Additionally, investigations should include the development of feedback mechanisms. Overall, more research is needed to establish the reliability of dCBT-I in routine clinical use.

## Conclusions

Shift workers are a vital component of a functioning society, and an occupational hazard of shift work includes insomnia. CBT-I is a proven effective treatment for insomnia. However, for shift workers suffering from shift work sleep disorder, there are barriers to accessing the traditional in-person format of CBT-I. The reviewed studies showed evidence that dCBT-I improves sleep quality in shift workers and is as effective as traditional in-person delivered CBT-I. Furthermore, it offers benefits such as improved emotional well-being, especially reduced symptoms of anxiety and depression. Given this evidence, shift workers suffering from shift work sleep disorder can use this more accessible version of dCBT-I to mitigate insomnia, anxiety, and depression symptoms. Healthcare systems employing shift workers should consider incorporating dCBT-I in employee wellness programs. Future studies with larger sample sizes, long-term follow-up, and more robust research designs will allow more conclusive recommendations about dCBT-I for shift workers. As digital health interventions continue to evolve, creating a more interactive and personalized digital experience may improve adherence and maximize the benefits shift workers receive from dCBT-I.
